# Central Autonomic Mechanisms Involved in the Control of Laryngeal Activity and Vocalization

**DOI:** 10.3390/biology13020118

**Published:** 2024-02-13

**Authors:** Marta González-García, Laura Carrillo-Franco, Carmen Morales-Luque, Marc Stefan Dawid-Milner, Manuel Víctor López-González

**Affiliations:** 1 Department of Human Physiology, Faculty of Medicine, University of Málaga, 29010 Málaga, Spain; laura_carrillo@uma.es (L.C.-F.); carmen6@uma.es (C.M.-L.); msdawid@uma.es (M.S.D.-M.); manuelvictor@uma.es (M.V.L.-G.); 2 Unit of Neurophysiology of the Autonomic Nervous System (CIMES), University of Málaga, 29010 Málaga, Spain; 3 Biomedical Research Institute of Málaga (IBIMA Plataforma BIONAND), 29010 Málaga, Spain

**Keywords:** central nervous system, laryngeal motor cortex, periacueductal gray matter, parabrachial complex, nucleus ambiguous, nucleus retroambiguus, laryngeal motoneurons, vocal emission, speech, laryngeal dystonia

## Abstract

**Simple Summary:**

In this review, the endeavor is to compile the most significant findings related to the interconnection among various autonomic centers that regulate the autonomic activity of the central nervous system. These centers appear to play a crucial role in the control of vocal emissions in mammals, including humans. Specifically, the aim is to comprehend and delineate the intricate neural networks involved in this functional relationship. This will allow us to describe how these structures, traditionally associated with cardiorespiratory control, also play a crucial role in the regulation of vocalization.

**Abstract:**

In humans, speech is a complex process that requires the coordinated involvement of various components of the phonatory system, which are monitored by the central nervous system. The larynx in particular plays a crucial role, as it enables the vocal folds to meet and converts the exhaled air from our lungs into audible sounds. Voice production requires precise and sustained exhalation, which generates an air pressure/flow that creates the pressure in the glottis required for voice production. Voluntary vocal production begins in the laryngeal motor cortex (LMC), a structure found in all mammals, although the specific location in the cortex varies in humans. The LMC interfaces with various structures of the central autonomic network associated with cardiorespiratory regulation to allow the perfect coordination between breathing and vocalization. The main subcortical structure involved in this relationship is the mesencephalic periaqueductal grey matter (PAG). The PAG is the perfect link to the autonomic pontomedullary structures such as the parabrachial complex (PBc), the Kölliker–Fuse nucleus (KF), the nucleus tractus solitarius (NTS), and the nucleus retroambiguus (nRA), which modulate cardiovascular autonomic function activity in the vasomotor centers and respiratory activity at the level of the generators of the laryngeal-respiratory motor patterns that are essential for vocalization. These cores of autonomic structures are not only involved in the generation and modulation of cardiorespiratory responses to various stressors but also help to shape the cardiorespiratory motor patterns that are important for vocal production. Clinical studies show increased activity in the central circuits responsible for vocalization in certain speech disorders, such as spasmodic dysphonia because of laryngeal dystonia.

## 1. Introduction

Communication in humans is exceptionally complex, encompassing cultural and social aspects, while communication in animals is primarily linked to survival and reproduction. While both share the ability to convey information, the form and scope of this communication vary significantly. Several concepts related to this communication process are important to distinguish. Phonation refers to the process of producing sounds through the vibration of vocal folds to create basic sounds. Vocalization, on the other hand, involves the articulation or production of specific sounds to communicate, representing a combination of sounds. Finally, speech is the most comprehensive and complex expression of language, encompassing meaningful communication through words and phrases, representing the highest level in the hierarchy of vocal communication.

Vocalization in animals is a complex phenomenon involving an intricate network of brain connections that coordinate the production of characteristic sounds. The ability to make sounds and communicate is crucial for most species, including humans. Communication in mammals is essential for transmitting information related to reproduction, location, identity, and the presence of predators, making it a survival-linked activity. When discussing the ability of some mammal species to develop vocalizations, it is important to consider that the structures and mechanisms they employ for this purpose are diverse and vary significantly [1]. 

In mammals, the main structure that allows phonation is the larynx, which is a stable structure with moving parts that, in humans, is composed of six cartilages, three of which are paired. In rats, another cartilage, the laryngeal alar cartilage, has been identified. While both humans and rodents utilize the larynx for vocalizations, there are differences in their vocalization mechanisms. Rats and mice possess muscles and cartilages not found in humans, enabling them to produce ultrasounds [2]. Despite these structural differences, there are similarities in the neural circuits involved in controlling phonation and both species share similar vocal fold properties due to their similar composition [3]. 

Laryngeal activity in rodents is controlled by the vagus nerve for intrinsic musculature and the hypoglossal nerve, pharyngeal plexus, and cervical nerves for extrinsic musculature. The vagus nerve, a component of the autonomic nervous system, is subject to cortical influence to some extent, allowing voluntary regulation of certain laryngeal functions such as phonation. It consists of the superior laryngeal nerve, whose external branch provides motor innervation to the cricothyroid muscle involved in vocal cord tension [4], and the internal branch offers somatosensory innervation to the glottis and the area above it for airway protection. The recurrent laryngeal nerve, another division of the vagus nerve, provides motor innervation to the remaining intrinsic laryngeal muscles, excluding the cricothyroid muscle, and sensory innervation to the upper trachea and lower glottis [5].

Within the human larynx, a distinction can be made between intrinsic and extrinsic musculature. The intrinsic muscles influence the opening and closing dynamics of the vocal folds as well as their elongation or contraction during phonation. Conversely, the extrinsic muscles are responsible for mobilizing the larynx for its ascent or descent. Intrinsic musculature includes the cricothyroid muscle, thyroarytenoid muscle, posterior and lateral cricoarytenoid muscles, and transverse and oblique arytenoid muscles. In rats, two bellies of the thyroarytenoid muscle (lateral and medial), an alar cricoarytenoid muscle, and a superior cricoarytenoid muscle have also been identified [2].

**Figure 1:**
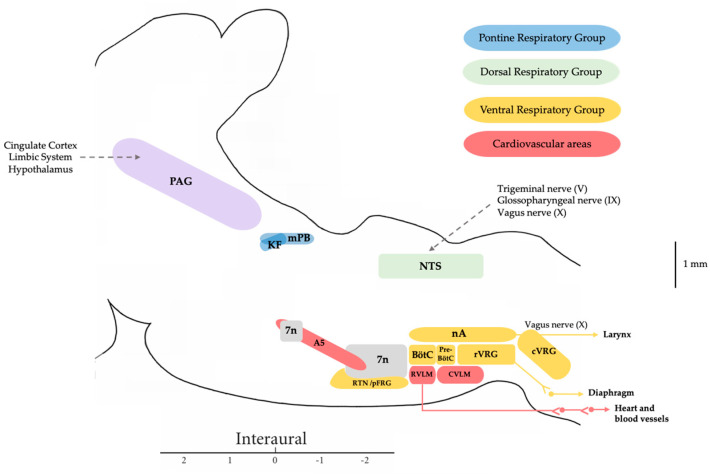
Diagram of the main mesencephalic and pontomedullary regions involved in cardiorespiratory and laryngeal control. Periaqueductal grey matter (PAG) modulates the ongoing activity in the pontomedullary respiratory circuits in response to behavioral and cognitive information; the pontine respiratory group (PRG) includes the Kölliker–Fuse nucleus (KF) and medial parabrachial (mPB) and it is involved in the control of the switch off between inspiration and expiration; the dorsal respiratory group (DRG) includes the nucleus of the solitary tract (NTS), it receives sensory information from the lungs and chemoreceptors and it is involved in the control of inspiration; the ventral respiratory group (VRG) includes the pre–Bötzinger complex (Pre–BötC) involved in the generation of rhythmic breathing, the Bötzinger complex (BötC) with expiratory neurons, the retrotrapezoid nucleus and parafacial respiratory group (RTN/pFRG) with chemosensitive neurons, the nucleus ambiguous (nA) where the laryngeal motoneurons are located, the rostral ventral respiratory group (rVRG) with inspiratory neurons, and the caudal ventral respiratory group (cVRG) with expiratory neurons. The A5 region, rostro–ventro–lateral medulla (RVLM), and caudal–ventral–lateral medulla (CVLM) are regions that are involved in cardiovascular control.

Various aspects of vocalization have been studied in different mammals, from rats to apes, aiming to better understand the development of speech in humans. Vocalizations require coordination between phonation and respiration, involving a large neural network that includes both anterior brain regions and the brainstem [6,7,8] (Figure 1).

In mammals, the respiratory system not only supplies the necessary oxygen to meet metabolic demands throughout life but also plays a fundamental role in the entire process of vocalization [9]. The muscles of the rib cage provide the force necessary for inspiration, with the diaphragm and external intercostal muscles serving as the main inspiratory muscles [10,11,12]. Expiration typically occurs passively when these inspiratory muscles relax, allowing air to be expelled. However, during activities such as vocalization, expiration can become an active process when the expiratory muscles of the abdominal region, specifically the internal intercostal muscles and abdominal muscles, are activated [10,12,13]. The respiratory system acts as a bellows, with its musculature controlled by motor neurons in the ventral horn of the thoracic and upper lumbar spinal cord. The diaphragm, for example, is controlled by the motoneurons of the phrenic nucleus. Impairment of the functionality of these muscles can disrupt the airflow process, affecting activities such as breathing, phonation, and speech [14,15,16]. In this airflow regulation, the laryngeal muscles also play a significant role. They expand to allow the passage of air into the lungs at the onset of inspiration, while at the end of this inspiration, they contract to decrease the airflow increasing expiratory time [17].

The rhythmic activity required to drive inspiration is carried out by a neural network located in different pontomedullary structures that allows phasic alternation between inspiration and expiration [18]. Vocal production, based on respiratory movements, means that the central circuits involved in vocalization are coupled to neurons generating respiratory patterns; in other words, the entire vocal circuit is integrated into the respiratory circuit. Furthermore, vocal emissions do not depend on normal breathing airflows but require higher air pressures to generate these sounds [19]. The regulation of airflow is achieved through muscles that adjust the resistance of the upper respiratory pathways.

## 2. Central Autonomic Network Involved in the Control of Respiration and Vocal Emission

A precise sequential contraction of the respiratory muscles is crucial and it requires control from different nuclei located in the brainstem. This control is necessary to optimize airflow by adapting muscular control to meet respiratory or vocalization requirements [20]. The vocalization in humans shares a common physiological and acoustic foundation with the vocal emission of other vertebrates. However, in humans, enhanced neural control of laryngeal muscles contributes to the stability of oscillations in the laryngeal vocal folds, regulating airflow from the lungs [6]. This enhanced stability in human phonation is attributed to an evolutionary reduction in anatomical complexity, such as the loss of structures like laryngeal sacs or vocal membranes present in non-human primates [21].

The coordination of vocalization processes is orchestrated by the central nervous system, with an intricate network extending from the laryngeal motor cortex to mesencephalic and pontomedullary regions. Key structures involved in the central control of vocalization include the periaqueductal grey matter (PAG), parabrachial complex (PBc), and Kölliker–Fuse nucleus (KF), nucleus ambiguus (nA), and nucleus retroambiguus (nRA). Subsequent research found that bilateral lesions in the PAG caused mutism, not only in animals but also in humans [6,22,23,24]. Studies on cats confirmed the importance of the caudal half of the mesencephalon and lesions in the PAG in vocal emission. Stimulations of the PAG and nRA produce vocal emissions, with observed neuronal activity within PBc during vocalization [25,26].

**Figure 2:**
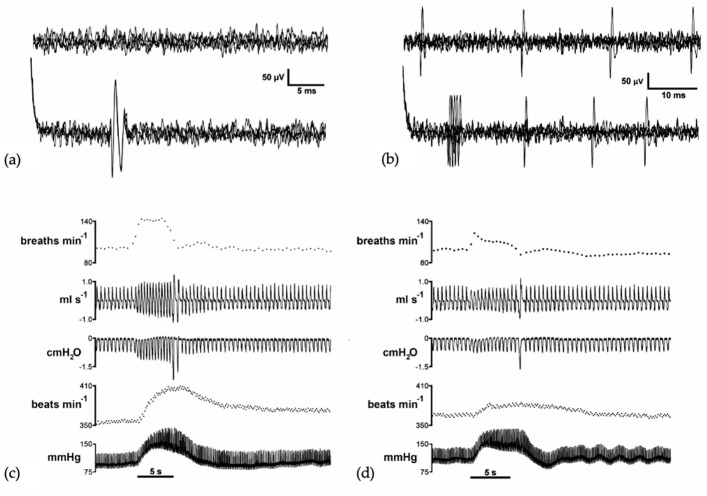
Extracellular recordings of three putative cells recorded from the A5 region. (**a**) Silent neuron (**upper** trace, four superimposed sweeps). The **lower** trace shows constant latency responses (four superimposed sweeps) to the dlPAG stimulation. (**b**) Spontaneously active cell (**upper** trace, five superimposed sweeps). The **lower** trace shows excitations with short latency responses from dlPAG stimulation (five superimposed sweeps). Instantaneous respiratory rate (**upper** trace, rpm), respiratory flow (mL/s), pleural pressure (cmH_2_O), instantaneous heart rate (bpm), and blood pressure (mmHg) in a spontaneously breathing rat showing the cardiorespiratory response evoked on dlPAG stimulation (**c**) before the microinjection of muscimol in the A5 region (50 nL over 5 s) and (**d**) after the microinjection of muscimol in the A5 region (50 nL over 5 s). Black line shows the onset of the dlPAG electrical stimulation (5 s). Authors’ figure modified from [32].

Additionally, we and others have demonstrated the role of several pontine nuclei in the control of the sympathoexcitatory response evoked from hypothalamic and mesencephalic regions in rats. Specifically, we have shown how both the PB-KF complex and A5 area maintain a functional relationship and modulate the defence responses evoked by the dorsomedial hypothalamic nucleus and PAG stimulation, with glutamate as the neurotransmitter involved [27,28,29]. It was shown that a blockade of the lateral parabrachial (lPB) and A5 area attenuated or abolished the cardiorespiratory response evoked from the dorsomedial hypothalamic nucleus or dorsolateral periaqueductal grey matter (dlPAG), while the medial parabrachial region and Kölliker–Fuse (mPB-KF) only mediated the cardiovascular response [30,31,32] (Figure 2). 

**Figure 3:**
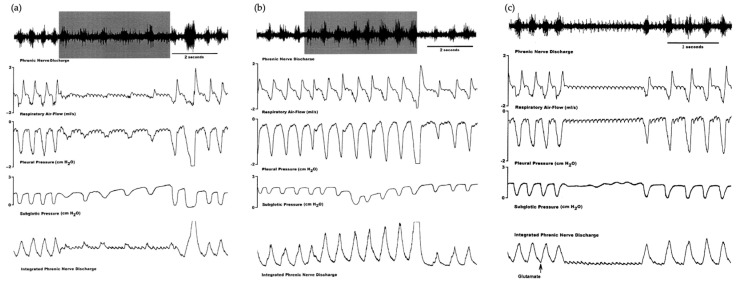
Laryngeal and respiratory responses to (**a**) electrical stimulation in the mPB (**b**) electrical stimulation in the lPB and (**c**) glutamate microinjection in the A5 region. Phrenic nerve discharge, respiratory airflow, pleural pressure, subglottic pressure, and integrated phrenic nerve discharge show an expiratory facilitatory response with an increase in subglottic pressure during electrical stimulation (20 μA, 0.4 ms pulses, 50 Hz for 5 s) in the medial parabrachial nucleus, an inspiratory facilitatory response with a decrease in subglottic pressure during electrical stimulation (10 μA, 0.4 ms pulses, 50 Hz for 5 s) in the lateral parabrachial nucleus, and an expiratory facilitatory response with an increase in subglottic pressure during a glutamate injection (10 nL over 5 s) in the A5 region. The arrow shows the onset of the injection. Authors’ figure modified from [33].

This dichotomy in the modulation of the respiratory response between mPB-KF and lPB was reflected in another study whereby lPB activation modified the respiratory and laryngeal responses, resulting in an increase in subglottic pressure and a decrease in respiratory rate and phrenic nerve activity. Conversely, activation of the mPB-KF and A5 area resulted in the opposite response, decreasing subglottic pressure and increasing respiratory rate and phrenic nerve activity [33] (Figure 3). This assigns an important role to these structures not only at the sympathoexcitatory level but also in laryngeal and respiratory control, as they can modify subglottic pressure levels and adjust them to the animal’s respiratory requirements. The aforementioned fits in perfectly with the historical role assigned to the mPB-KF in respiratory control, referred to as the pneumotaxic center. Actually, both constitute the pontine respiratory group (PRG), which is involved in the switch-off from inspiration to expiration through the reciprocal connections with the dorsal respiratory group (DRG) and ventral respiratory group (VRG) within the medulla oblongata [34]. The rhythmic activity required to drive inspiration is carried out by a neural network located within the pre-Bötzinger complex (pre-BötC) [18], which is triggered in the inspiration phase and indirectly drives the inspiratory muscles [9,35]. This inspiration is also determined by the activation of the KF [36]. Finally, the lateral parafacial nucleus is activated during active expiration [9].

In the following, we will describe the different contributions of these autonomic regions to the central control of respiration and vocal emission. 

### 2.1. Periaqueductal Grey Matter

The PAG is a structure located in the mesencephalic region of the brainstem, playing a fundamental role in regulating and integrating responses related to defensive behavior in mammals, their autonomic response, and the processing of painful stimuli. It is a highly interconnected region, both anatomically and functionally, with the anterior brain and the pons. Its primary function involves integrating behavioral responses to internal and external stimuli, making it an area involved in respiratory and cardiovascular responses [29,32,37]. 

The PAG receives a significant number of afferents, originating from the prefrontal cortex, amygdala, and hypothalamus. Its efferents, in turn, project to pontine nuclei, allowing for the coordination of patterns in cardiorespiratory and motor responses in response to stimuli. Beyond its role in vocalization, the PAG serves additional functions such as thermoregulation, the regulation of sleep-wake cycles, and the modulation of neuropathic pain or micturition. In the clinical context, alterations in the activity of this area have been observed in conditions such as Alzheimer’s disease or multisystem atrophy [38,39].

The PAG lacks well-defined cellular groups; however, functional attributions can be approximated to about four longitudinal columns distinguished by their distinct connectivity patterns. Studies conducted in primates and rodents demonstrated that the PAG can be divided into dorsomedial, dorsolateral, lateral, and ventrolateral subregions, each exhibiting distinct chemical, functional, and connectivity properties [38,40,41]. Furthermore, the PAG is subdivided into different columns, each activating distinct responses. The ventrolateral column is responsible for defensive behaviors, hypersensitivity, hypotension, bradycardia, and opioid-mediated analgesia. In contrast, the dorsal column promotes active coping and fighting behaviors, hypertension, tachycardia, and non-opioid analgesia [42].

The PAG plays a crucial role in the regulation and integration of defensive behaviors, autonomic responses, and the processing of painful stimuli in mammals. In the respiratory domain, the PAG is associated with the integration and adaptation of respiratory responses under different conditions. The stimulation of dlPAG and lateral periaqueductal grey matter (lPAG) induces tachypnea and they are mostly involved in active coping strategies, such as fighting or fleeing, while the stimulation of dorsomedial PAG and ventrolateral periaqueductal grey matter (vlPAG) elicited bradypnea, profound respiration, dyspnea, and inspiratory apneas that it is associated with passive coping strategies, such as freezing [29,32,41,42]. These responses are mediated by projections to premotor interneurons in the pons and the nRA in the medulla [38,43]. 

The PAG area serves as a crucial hub between the brainstem and midbrain, receiving inputs from various cortical areas as well as the lateral, dorsomedial, and paraventricular hypothalamus [44,45]. Ascending input to the PAG comes directly from the spinal cord, particularly the upper cervical spinal cord, projecting to the lPAG and vlPAG [46]. Additionally, the nucleus tractus solitarius (NTS) projects to the vlPAG and medial PAG [47]. Other respiratory centers in the brainstem also project to the lPAG and vlPAG, including the pre-BötC and Bötzinger complex (BötC) complexes, KF [35,44], locus coeruleus [48], lPB and the medial parabrachial region (mPB) [49], the paratrigeminal nucleus [50], and cerebellar fastigial and dentate nuclei [51]. In this context, the PAG assumes a pivotal role in effectively coordinating limbic, corticoprefrontal, and cingulate afferents, thereby facilitating the requisite modification of activity in mesencephalic-pontomedullary nuclei responsible for laryngeal control [52,53].

The PAG exerts its influence over the pontomedullary respiratory nuclei involved in cardiorespiratory rhythmogenesis, orchestrating the transition from eupnea to a rhythm tailored to vocalization or grunting. This tailored rhythm is essential for vocal and expressive activities. Precise control over the pattern and intensity of motor neuron activation in respiratory, laryngeal, oropharyngeal, and craniofacial structures is crucial [52,54]. Successful vocalization necessitates a meticulous and prolonged expiration to establish adequate and sufficient airflow pressure, ensuring a subglottic pressure conducive to vocalization [53]. Specifically, nRA emerges as a prime target for converting passive respiration into active respiration, instigating motor activities that impact abdominal pressure. Furthermore, it modulates the activity of motoneurons located in the nA, responsible for controlling the caliber of the pharynx and larynx [53,55].

### 2.2. Pontine Nuclei

As demonstrated, respiratory rhythmogenesis is orchestrated by circuits involving the caudal region of the brainstem. Nevertheless, anterior areas also play a pivotal role in the indispensable respiratory modulation for vocalization. The PB-KF complex, also considered PRG, is recognized as a central component within these respiratory circuits.

Firstly, the KF is an area that exerts influence over the transition from inspiration to expiration [56]. Neurons in this nucleus exhibit activity linked to specific phases of respiration, remaining active during inspiration and abruptly ceasing firing at the termination of inspiration [56,57]. This area modulates the activity of the phrenic nucleus [58], as well as the nA, hypoglossal, and facial nuclei [58,59,60], enabling premotor neurons of the KF nucleus to activate muscles involved in airflow regulation, leading to airflow reduction during post-inspiration [61]. The KF primarily gates the post-inspiratory phase [58,62] and, in addition, its activity is strongly associated with vocalization in mammals as well as swallowing, tongue movements, and larynx/pharynx control [63]. Respiratory and cardiac coordination occurs in a cyclical manner, wherein during the inspiration, the heart rate increases and during post-inspiration and active expiration, it decreases again [64,65]. The KF nucleus provides the phasic excitatory drive to these cardiac premotor neurons to produce a respiratory sinus arrhythmia [66]. This arrhythmia, due to these respiratory-related changes, is produced by the activity of inhibitory preganglionic parasympathetic cardiac vagal neurons, which are located primarily in the nA [67,68]. 

Secondly, the PBc is a brain region interconnected with the PAG and is involved in the regulation of cardiovascular and respiratory functions. The PBc can be divided in different subdivisions. The lPB and mPB serve as integrative regions at the pontine level, helping to establish the duration of inspiration and expiration [69]. The medial subdivision works in conjunction with the KF nucleus. The mPB contains more expiratory neurons than the KF nucleus, which has more inspiratory and phase neurons [70]. Therefore, the mPB’s main function is to modulate the duration of expiration [71]. Furthermore, PBc functions as an intermediate nucleus that projects to the laryngeal motor cortex and the nA [25,58]. Studies conducted in monkeys, utilizing extracellular recordings, have demonstrated the presence of active neurons in the PBc during vocal emissions [25,70]. Additionally, the induction of species-typical calls was observed with the stimulation of areas near the PBc [72] The PBc exerts control over the respiratory duration, a function attributed to somatosensory feedback from the lungs and larynx, transmitted through the NTS and other nuclei [25,57]. This regulatory role in respiratory phases strongly suggests its indispensable role in mammalian vocalization due to PBc projections to motor nuclei such as the nA and to nuclei of the V and XII cranial nerves [60]. 

### 2.3. Medullary Nuclei

Brainstem regions within the medulla oblongata and pons contain neuron clusters crucial for ventilation regulation. The VRG and DRG are placed within the medulla oblongata, constituting the medullary respiratory center. The DRG functions as the “inspiratory center”, while the VRG predominantly serves an expiratory role.

The DRG is placed in the dorsomedial medulla within the ventrolateral NTS and predominantly houses inspiratory neurons. This specific subnucleus of the NTS serves as the location where afferent fibers from the glossopharyngeal and vagus nerves convey sensory information from peripheral chemoreceptors (monitoring blood gas levels) and lung mechanoreceptors (monitoring muscle and joint movement). The sensory feedback is transmitted to the DRG, where it forms synapses with premotor inspiratory neurons, the majority of which project to spinal motor neurons [73]. The DRG plays a role in sustaining a steady breathing rhythm by activating inspiratory muscles at regular intervals post-passive exhalation. It remains inactive during passive exhalation. An appropriately timed signal to inspiratory muscles results in a breathing rate typically ranging between 12–15 breaths per minute in humans. Additionally, it receives signals from the PRG, which receives information from chemoreceptors and mechanoreceptors in the body, along with signals from higher brain regions. This information is assessed and signals are then sent to the VRG, adjusting its ventilation actions—either increasing frequency or depth or decreasing them—based on muscle and body requirements [74].

The VRG comprises a ventrolateral column of respiratory neurons, encompassing the nA, nRA, pre-BötC, and BötC [9,18,75]. Within the VRG, both expiratory and inspiratory neurons are present, with their projections extending to other brainstem neurons or serving as premotor neurons projecting to respiratory motor neurons. Expiratory and inspiratory neurons are distinctly localized in the caudal ventral respiratory group (cVRG) and rostral ventral respiratory group (rVRG) for expiration and intermediate VRG for inspiration, respectively. The caudal segment of the VRG, along with the nearby BötC, forms the “expiratory center” [9].

#### 2.3.1. Pre-Bötzinger and Bötzinger Complexes

The Pre-BötC provides the rhythmic activity necessary to drive inspiration [18]. These neurons fire in the inspiratory phase and indirectly drive the inspiratory muscles [35]. Their activity depends on the O_2_ and CO_2_ pressures in the bloodstream, as well as the situation in the chest cavity. This information is transmitted to the central nervous system via the vagus nerve to the lateral NTS, where the afferent fibers of the vagus nerve terminate [76]. Although it presents direct projections to multiple respiratory motor nuclei, indirect projections appear to be more common. It modulates to the phrenic nucleus via neurons located in the rVRG [16] and to thoracic motor neurons controlling abdominal muscles via the cVRG [35].

The BötC, located at the most rostral part of the VRG, houses neurons that inhibit most inspiratory neurons during expiration, playing a significant role in expiration control [77]. The BötC includes inhibitory neurons, characterized by GABAergic and glycinergic properties, exhibiting decrementing (post-inspiratory, post-I) or augmenting (aug-E) firing patterns during expiration [78]. These neurons are believed to establish reciprocal synaptic interactions with pre-BötC neurons [77]. Suppression of BötC activity or disruption of inhibitory synapses in both BötC and/or pre-BötC significantly depresses breathing, underscoring the critical role of reciprocal inhibition between BötC and pre-BötC in respiratory rhythm regulation and the generation of eupnoeic breathing [79]. Consequently, it is proposed that BötC and pre-BötC neurons constitute the essential core of the respiratory network [80].

#### 2.3.2. Nucleus Ambiguus

The cell bodies of neurons that innervate the intrinsic muscles of the larynx are located in nA, which is a column of neurons oriented rostrocaudally and situated in the ventrolateral portion of the medulla oblongata. It extends from the facial nerve motor nucleus to the pyramidal decussation [81]. The nA can be subdivided into three main components: the compact formation (housing motoneurons innervating the esophagus), the semi-compact formation (containing motoneurons innervating the pharynx and the cricothyroid muscle of the larynx, innervated by the superior laryngeal nerve), and the loose formation (comprising motoneurons innervating the laryngeal muscles, excluding the cricothyroid) [81,82].

The motoneurons located in the nA control the intrinsic musculature of the larynx (controlling the tension, length, and position of the vocal folds), while the extrinsic musculature (suprahyoid and infrahyoid musculature contributing to the movement and position of the larynx in the neck) is controlled by motoneurons extending from the hypoglossal nucleus to the ventral horn of the second cervical segment [83]. These motoneurons innervate the larynx through the vagus nerve. However, it is not only important to control the larynx but it is also necessary to control the other elements involved in vocal emission, such as subglottic pressure. Regarding breathing, the musculature is controlled by motoneurons from the ventral horn of the thoracic and upper lumbar spinal cord; and in the case of articulation, it is regulated by motoneurons from the facial and trigeminal nucleus, ventral horn of the upper cervical spinal cord, rostral nA, and hypoglossal nucleus [6]. 

#### 2.3.3. Nucleus Retroambiguus 

The nRA is a region of the brainstem that plays a fundamental role in the regulation of breathing. It is caudal to pre-BötC and it contains inspiratory and expiratory premotor neurons [84]. Research has indicated the presence of distinct groups of premotor neurons in the nRA. Experimental findings in cats reveal that activation of specific regions within the nRA induces vocalizations without concomitant alterations in inspiration. In contrast, stimulation in other areas of the nRA fails to elicit vocal emission but does increase respiratory frequency by abbreviating both inspiratory and expiratory durations. This suggests the existence of diverse neuronal subsets within the nRA governing both vocalization and respiratory functions [85]. 

Some authors consider this nucleus part of the VRG and subdivide it into either cVRG or rVRG [86], while others specifically refer to the nRA as the cVRG [85]. In this review, we decided to maintain the classification in rVRG and cVRG. 

The rVRG serves as an intermediary between the pre-BötC and the phrenic motor nucleus, providing input to diaphragmatic motor neurons [87]. This group is specifically associated with the inspiratory phase. According to recent research, these neurons may play a role in shaping the pattern of respiratory motor output, processing and transmitting sensory input information, and coordinating ventilation with motor activity by regulating the activity of respiratory muscles [88]. All of this is achieved through their projections to the nA, hypoglossal, and facial nuclei [8,89].

The cVRG is an area that houses expiratory premotoneurons [84], responsible for controlling any activity related to expiration, such as vocalization [85]. Vocalization activity depends on the projection of this area to the nA and spinal cord. This area innervates motoneurons of the abdominal muscles in the thoracic spinal cord and those of the muscles in the upper respiratory pathways in the nA, hypoglossal, trigeminal, and facial nuclei [90,91,92]. It also has outputs directed towards the pre-BötC and BötC, the KF, the rVRG, the retrotrapezoid nucleus, the lPB, and the PAG [92,93]. Regarding the afferents that cVRG receives, the primary input related to vocalization largely comes from the PAG [85,91]. It also receives inputs, both excitatory and inhibitory, from the pre-BötC [35] and the BötC, the KF, the lateral parafacial nucleus, and the rVRG [60,94]. It also receives direct inputs from the NTS [94], the retrotrapezoid nucleus [93,94], and the lPB and mPB [94].

## 3. Vocalization in Apes: Connectivity between the PAG and the Laryngeal Motor Cortex

Voluntary voice production in humans involves sound modulation and directly depends on the laryngeal motor cortex, situated in the dorsal portion of the ventral zone of the primary motor cortex. Its direct connection with laryngeal motor neurons of the nA/nRA governs the laryngeal muscles for learned vocal pattern emission [7]. However, it is demonstrated that during vocal emissions, there is concurrent activation of the voluntary and involuntary systems [95]. Automatic involuntary activation of the pathway originating in the primary motor cortex and passing through the PAG and cVRG is necessary to impart appropriate emotional character to vocal emissions. This necessitates the activation of the pathway from the laryngeal motor cortex directly to corticomedullary fibers, activating motor neurons for facial, mouth, tongue, larynx, and pharynx control for word and phrase production [52].

In apes, the exploration of pathways governing voluntary and involuntary vocalizations has developed a model that explicates vocal control through two hierarchically organized and related networks. Involuntary vocalizations, such as crying or laughing, are regulated by limbic mechanisms distinct from those governing voluntary vocalizations or speech [6]. These emotional expressions are directed by the emotional system, comprised of specific pathways targeting the brain stem and spinal cord [95,96].

Results obtained from the squirrel monkey suggest that the system includes the cingulate gyrus, PAG, and various pontine and medullary nuclei [97]. Vocal control ultimately hinges on the primary motor area, a bilateral structure responsible for laryngeal control and orofacial musculature [98], alongside activation of the superior temporal gyrus to compensate for alterations in auditory feedback during phonation. Two feedback loops, involving the basal ganglia and cerebellum, provide the motor cortex with the necessary information for executing motor commands in phonation. However, these structures appear unnecessary to produce innate vocal patterns. Hence, in humans, the neural system that is involved in emotional expressions like laughter and crying includes different regions from the anterior cingulate cortex, the PAG, nRA, and nA, yet its contribution appears less pivotal in deliberate expressions such as voice and speech, where cortical control seems to exert greater influence [99]. Furthermore, the speech production system involves predominant activation of the left hemisphere, encompassing the superior temporal gyrus, anterior insula, basal ganglia, and cerebellum. For this production, the activity of the cingulate gyrus and PAG is also necessary, to varying degrees, to associate emotional character with vocal production [100].

The PAG receives projections from upper limbic regions and cortical areas, including the anterior cingulate gyrus, insula, and orbitofrontal cortex. It maintains connections with the cVRG, which, in turn, has direct access to motor neurons governing vocalization. Specifically, it controls motor neuron groups governing the soft palate, pharynx, larynx, diaphragm, intercostal, abdominal, and pelvic muscles. The primary objective is to regulate/modify intra-abdominal, intrathoracic, and subglottic pressure, critical for vocalization generation [48,86,91]. In apes, vocalizations, in addition to PAG activation, can be elicited by electrical stimulation of various brain regions, such as the hypothalamus, amygdala, bed nucleus of the stria terminalis, orbitofrontal cortex, and anterior cingulate gyrus, all strongly connected to the PAG. The prerequisite is the integrity of the PAG [101]. Contrarily, stimulation of areas not connected to the PAG, such as the motor or premotor cortex, fails to induce vocalizations [102], underscoring the pivotal role of the PAG in vocalization in primates and humans. The coordination extends to partial vocalizations generated by activating caudal PAG levels through its connection with the cVRG [54,85,103].

## 4. Clinical Implications

All these central structures described above share the commonality of mediating autonomic responses to environmental stress and supporting vocalization. Recent studies demonstrate that laryngeal microstructure and its innervation undergo similar changes during development in rodents and humans [104]. Central circuits responsible for vocalization exhibit deregulation in certain central speech disorders, such as spasmodic dysphonia due to laryngeal dystonia, paradoxical laryngeal adduction movements, or muscle tension dysphonia between others. A better description and understanding of the individual contribution of each nucleus in the mentioned network on the central control of laryngeal motoneurons would not only enhance our understanding of normal phonatory control but also contribute to a better understanding of central alterations occurring in this type of vocal-related disorders that are described in more detail below.

Specifically, laryngeal respiratory apnea often constitutes a clinically severe manifestation, as seen in newborn apnea or central sleep apneas, caused by immaturity or abnormalities in the central respiratory control in individuals, leading to an exaggerated response of the laryngeal adduction reflex [105,106]. Moreover, it is also known that spasmodic dysphonia, a focal form of dystonia, is a neurological voice disorder characterized by involuntary “spasms” of the vocal folds, resulting in speech interruptions and affecting voice quality. The cause of spasmodic dysphonia is unknown, although there is some consensus that it involves a central nervous system alteration, particularly in motor control, as alterations in the basal ganglia, cerebellum, and sensorimotor cortex circuitry have been described, along with structural changes in corticobulbar and corticospinal tracts, which are the nerve tracts in contact with bulbar neurons responsible for phonation [107]. Paradoxical laryngeal adduction movements are characterized by the adduction or approximation of the vocal folds during the respiratory cycle (especially during the inspiratory phase), causing obstruction of the laryngeal airway. The resulting dyspnea and stridor are often confused with asthma but do not respond to treatment with steroids and bronchodilators, as glottic narrowing is independent of bronchial lumen caliber. The origin of this intermittent interruption of transglottic airflow due to paradoxical laryngeal adduction remains to be elucidated. It has been associated with laryngeal irritation by agents such as gastroesophageal reflux or acute severe stress [108]. Muscle tension dysphonia corresponds to a vocal disorder provoked by inappropriate laryngeal muscle use. This pathology involves increased muscle tension in the larynx and, more specifically, insufficient relaxation of the posterior cricoarytenoid muscle controlled by the nA (abductor of the vocal folds) during the phonation process. There is also an imbalance of synergistic and antagonistic muscle forces, the persistence of which produces organic alterations at the level of the vocal folds, exacerbating the condition associated with stress [109].

## 5. Conclusions

Vocalization in mammals is underpinned by an intricate neural network. This comprehensive review of the neuronal circuits involved in laryngeal and respiratory control has provided a thorough understanding of the complexity and interconnection of these neural networks. From the activation of key regions in the midbrain and brainstem to finely tuned coordination with supraspinal areas and peripheral sensory feedback, we have delineated the intricate mechanisms regulating vocalization and respiration.

The main subcortical structure involved in this relationship is the PAG. This region is the perfect link to the autonomic pontomedullary structures that modulate both cardiorespiratory autonomic function and vocalization. Anterior areas of the brainstem that also play a pivotal role in the indispensable respiratory modulation for vocalization are the PB-KF complex, also considered as PRG. The control of these structures over the medullary circuits that subserved respiratory rhythmogenesis is crucial for the development of an adequate vocalization. This respiratory rhythmogenesis is orchestrated by two different groups of medullary nuclei: the VRG, with a predominant expiratory role, and the DRG, considered as an inspiratory center. Regarding vocalization, the relationship between the premotoneurons located within the cVRG and the motoneurons within the nA and the control that PAG and other pontomedullary nuclei exert on these areas provide the neural substrate to regulate all the elements involved in vocal emission.

Ultimately, this review mentioned different vocal disorders related with central alterations of this neural circuit, thereby laying the groundwork for future research aimed at deciphering the molecular and cellular mechanisms underlying these neural networks. Our understanding of these complex interactions enables more precise and personalized therapeutic approaches to enhance the quality of life for those patients affected by these disorders.

## Data Availability

No new data were created or analyzed in this study. Data sharing is not applicable to this article.

## Abbreviations

BötC	Bötzinger complex
cVRG	caudal ventral respiratory group
dlPAG	dorsolateral periaqueductal grey matter
DRG	dorsal respiratory group
KF	Kölliker–Fuse nucleus
LMC	laryngeal motor cortex
lPAG	lateral periaqueductal grey matter
lPB	lateral parabrachial
mPB-KF	medial parabrachial region and Kölliker–Fuse nucleus
mPB	medial parabrachial
nA	nucleus ambiguus
nRA	nucleus retroambiguus
NTS	nucleus tractus solitarius
PAG	periaqueductal grey matter
PB	parabrachial
PBc	parabrachial complex
Pre-BötC	pre-Bötzinger complex
PRG	pontine respiratory group
rVRG	rostral ventral respiratory group
vlPAG	ventrolateral periaqueductal grey matter
VRG	ventral respiratory group
